# Biased gene transfer and its implications for the concept of lineage

**DOI:** 10.1186/1745-6150-6-47

**Published:** 2011-09-23

**Authors:** Cheryl P Andam, J Peter Gogarten

**Affiliations:** 1 Department of Molecular and Cell Biology, University of Connecticut, 91 North Eagleville Road, Storrs, CT 06269-3125 USA

## Abstract

**Background:**

In the presence of horizontal gene transfer (HGT), the concepts of lineage and genealogy in the microbial world become more ambiguous because chimeric genomes trace their ancestry from a myriad of sources, both living and extinct.

**Results:**

We present the evolutionary histories of three aminoacyl-tRNA synthetases (aaRS) to illustrate that the concept of organismal lineage in the prokaryotic world is defined by both vertical inheritance and reticulations due to HGT. The acquisition of a novel gene from a distantly related taxon can be considered as a shared derived character that demarcates a group of organisms, as in the case of the spirochaete Phenylalanyl-tRNA synthetase (PheRS). On the other hand, when organisms transfer genetic material with their close kin, the similarity and therefore relatedness observed among them is essentially shaped by gene transfer. Studying the distribution patterns of divergent genes with identical functions, referred to as homeoalleles, can reveal preferences for transfer partners. We describe the very ancient origin and the distribution of the archaeal homeoalleles for Threonyl-tRNA synthetases (ThrRS) and Seryl-tRNA synthetases (SerRS).

**Conclusions:**

Patterns created through biased HGT can be undistinguishable from those created through shared organismal ancestry. A re-evaluation of the definition of lineage is necessary to reflect genetic relatedness due to both HGT and vertical inheritance. In most instances, HGT bias will maintain and strengthen similarity within groups. Only in cases where HGT bias is due to other factors, such as shared ecological niche, do patterns emerge from gene phylogenies that are in conflict with those reflecting shared organismal ancestry.

**Reviewers:**

This article was reviewed by W. Ford Doolittle, François-Joseph Lapointe, and Frederic Bouchard.

## Background

Horizontal gene transfer (HGT) has had a tremendous impact on how evolution has shaped life's history, which, since Darwin, is generally described as the Tree of Life. Instead of a strictly furcating tree representing vertical inheritance, numerous horizontal transfers have resulted in a reticulated evolutionary history. Hence, the genealogical history of a genome cannot be portrayed simply as a lineal descent from a common ancestor that existed in some distant past because a lineage can trace its ancestry from a myriad of sources, both living and extinct.

By introducing novel genetic material into a genome, the process of HGT can accomplish massive leaps through character space, endowing organisms with novel metabolic processes and physiological capacities that would have otherwise taken millions of years for lineages to acquire through mutations [1]. For example, a recent study reported that the genes involved in the degradation of an algal polysaccharide were transferred via seaweed consumption from marine bacteria to the gut bacterium *Bacteroides *isolated from Japanese individuals [2]. The massive gene exchange observed in *Shewanella baltica *isolates includes many genes involved in anaerobic metabolism, thus allowing them to successfully adapt to the deeper, anoxic waters of the Baltic Sea [3]. Adaptation of *Prochlorococcus *to phosphate limitation was made possible through horizontal transfer of phosphate acquisition genes [4]. Gene transfer, and not gene duplications, appears to be the primary driving force in the expansion of gene families in prokaryotes [5,6]. HGT can therefore be exploited as an adaptive strategy by many organisms, allowing them to take advantage of new environments and/or metabolic compounds that may provide them a competitive benefit over others. Although HGTs contribute to bacterial adaptation by bringing in novel genes into the genome, the majority of transfers may be nearly neutral to the recipient, thus impacting the gene content without conferring any immediate benefits to the recipient organisms [7]. Some of these horizontally acquired genes, however, can be considered as a pre-adaptation, which the recipient can potentially use.

Horizontal transfer events are generally a result of the proximity of donor and recipient lineages inhabiting the same ecological niche, with genetic material being transferred over short physical distances [8] without regard to domain boundaries. High frequency of HGT has been observed in marine microbial populations, providing flexibility in their genetic composition and allowing them to easily adapt to changing environmental conditions [9]. The close proximity of a diverse array of microbes in the gut provides an ideal hot spot for HGT, as in the case of the transfer of sulphonamide reisistance genes between strains of *Escherichia coli *residing in the human intestine [10]. Prokaryotes that inhabit high-temperature environments -- an ecological niche shared by divergent organisms -- provide an example of highways of gene sharing [11] that dominate the phylogenetic information content present in a genome [12,13]. Microbial consortia in mixed biofilms also facilitate horizontal acquisition of chromosomal DNA and can lead to the spread of antibiotic resistance genes among the microbial inhabitants [14].

In this article, we explore the role of HGT in defining and shaping prokaryotic lineages. Gene transfer is often viewed as creating conflicting relationships in microbial phylogeny, resulting to topological discrepancy between the gene trees and the species tree or organismal tree [15]. In the case of horizontal acquisitions from distant relatives, the gene of the recipient taxon would exhibit high similarity to the donor group, despite the evolutionary distance that separates them. On the other hand, when closely related partners in a group exhibit preferences for exchanging genes with one another, such sharing may eventually lead to cohesion of the group [16]. In view of the dominating role of HGT in prokaryotic evolution, we propose a re-evaluation of the concept of lineage in the microbial world.

## Results and Discussion

### Horizontally transferred PheRS as a shared derived character

**Figure 1:**
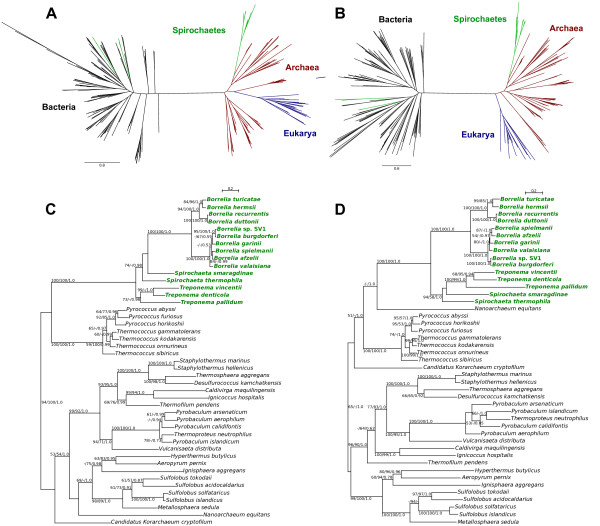
**Phylogenetic analyses of Phenylalanyl-tRNA synthetases (PheRS)**. Maximum likelihood phylogenetic tree of the (A) α subunit and (B) β subunit of PheRS across the three domains of life. A detailed look at the Spirochaete (C) PheRS- α and (D) PheRS-β subunits shows close relationships with the Archaea. Numbers on the nodes indicate bootstrap support under maximum likelihood (left) and distance (middle), and posterior probabilities (right) [59].

Aminoacyl-tRNA synthetases (aaRS) catalyze the attachment of tRNA with its cognate amino acid, crucial in maintaining the fidelity of the genetic code and the translation process. Unlike the majority of aaRS, PheRS exhibits a multidomain α_2_β_2 _heterotetrameric structure. Phylogenetic reconstruction of the PheRS sequences across the three domains of life reveal an archaeal origin of the PheRS harbored by the spirochaete genera *Spirochaeta, Borrelia *and *Treponema*, distinct from those found in other closely-related taxa, *Leptospira *and *Brachyspira*, which have the typical bacterial form (Figure 1A, B). The phylum Spirochaetes consists of three recognized families-Brachyspiraceae, Leptospiraceae and Spirochaeataceae, the latter group consisting of the genera *Borrelia, Spirochaeta *and *Treponema *(based on NCBI taxonomy). Spirochaetes are helically-shaped, gram-negative, motile bacteria classified in 13 genera. They represent one of the deeply branching clades in the bacterial domain. The phylogenies of each PheRS subunit present the same evolutionary picture of the spirochaete PheRS, suggesting that both subunits were horizontally acquired from Archaea to the common ancestor of *Spirochaeta, Borrelia *and *Treponema*. Both subunits of the archaeal form of the spirochaete PheRS show close affinities with *Thermococcus *and *Pyrococcus*, both members of the order Thermococcales in the phylum Euryarchaeota (Figure 1C, D). The β subunit also exhibits a close relationship with *Nanoarchaeum*, although this relationship has low bootstrap support (Figure 1D). This suggests that the two PheRS subunits were transferred together in a single event from the same Archaeal donor to the ancestor of these spirochaetes.

The archaeal and bacterial form of PheRS found in Spirochaetes show high divergence (26-29% identity for the α subunit, and 21-26% for the β subunit). The α subunit of *Spirochaeta, Borrelia *and *Treponema *range from 513-528 amino acids and the β subunit from 514-573 amino acids, which are in the range to those found in Thermococcales species (α subunit with 499-503 amino acids and β subunit with 556-574 amino acids). *Leptospira *and *Brachyspira*, on the other hand, possess an α subunit with a size of 339-342 amino acids and a β subunit with 807-808 amino acids, similar to those found in other Bacteria.

A previous study has reported the archaeal origin of PheRS in *Borrelia *and *Treponema *[17], many of which are pathogenic to humans. The major clinical diseases caused by these two genera include Lyme disease, relapsing fever and syphilis [18]. Thus, it initially came as a surprise that these human-associated pathogens have acquired their PheRS from extremely thermophilic organisms. The key to this remarkable inter-domain genetic connection is the genus *Spirochaeta*. This genus represents a group of saccharolytic, facultatively anaerobic or obligately anaerobic free-living spirochetes that thrive in a variety of aquatic environments. A number of species inhabit extreme environments, including *S. halophila *isolated from a high-salinity pond on the Sinai shore [19], *S. thermophila *from brackish and marine hot springs in New Zealand and Russia [20], *S. caldaria *from freshwater [21], *S. smaragdinae *from an African oil field [22], *S. americana *from alkaline, hypersaline Mono Lake [23], and *S. dissipatitropha *from anaerobic sediments of the alkaline, hypersaline Owens Lake in California [24]. Based on 16S rRNA relationships, *Spirochaeta *shows closest affinities with the host-associated genera *Treponema *and *Borrelia *[18]. In the PheRS phylogenies (Figure 1C, D), *Spirochaeta *is found at the base of the tree, with *Borrelia *and *Treponema *diverging later on. Hence, the most parsimonious hypothesis to explain the evolutionary trajectory of PheRS in spirochaetes is that the most recent common ancestor of *Spirochaeta, Borrelia *and *Treponema *was most physiologically similar to that of *Spirochaeta *in terms of its thermophilic requirement.

This spirochaete ancestor most likely inhabited environments in which *Pyrococcus *and *Thermococcus *thrived as well, resulting to the replacement of the bacterial PheRS that this ancient spirochaete originally possessed with an archaeal form. The archaeal PheRS was maintained in this lineage until its divergence into the three extant spirochaete genera. A species of *Spirochaeta, S. coccoides*, has recently been isolated from the hindgut of the termite *Neotermes castaneus *and it was suggested that they play an important role in the synergistic degradation of the main polymeric wood compounds, cellulose and hemicellulose [25]. Currently, most spirochaetes that are known to be symbiotically associated with termites are members of the genus *Treponema*. Hence, it is likely that the most recent ancestor of these three spirochaete lineages started out as a thermophile and eventually branched out into different ecological niches, including eukaryotic host organisms, where many now dwell either as mutualistic or pathogenic inhabitants.

Phenylalanine, along with tryptophan and tyrosine, are the only amino acids from the standard 20 that contain an aromatic ring. The possession of an archaeal PheRS in thermophilic organisms, including a spirochaete ancestor, can confer an advantage in organisms that inhabit extreme environments. Previous studies have reported that aromatic proteins and their interaction are known to influence structural stabilization of proteins [26], particularly important for thermophilic enzymes to maintain full activity at elevated temperatures [27] and prevent thermal denaturation [28]. Moreover, additional aromatic clusters were found to be present in many different thermophilic protein families, which were lacking in their mesophilic homologs [28]. Hence, thermophilic spirochaetes may have achieved increased viability during the course of their evolution by making use of an efficient PheRS acquired from thermophilic Archaea that could satisfy the increased demand for Phe incorporation into proteins to provide additional stability to their proteins.

Gene transfer has tremendously affected the formation of groups of organisms. Although horizontal transmission can be a challenge in defining phylogenetic relationships, the presence of a transferred gene can constitute a shared derived character (or synapomorphy) useful in classification and in the identification of monophyletic clades. A shared derived character is an evolutionary novelty unique to a particular clade and the acquisition of exogenous genetic material through HGT establishes the recipient and its descendants as a monophyletic group [29]. For example, the monophyly of the opisthokonts (which include animals and fungi) was confirmed through the horizontal transfer of Tyrosyl-tRNA synthetase (TyrRS) from the archaeal group Halobacteria to the opisthokont ancestor [30]. In the case of the spirochaetes, the transfer of PheRS to this ancient helical-shaped organism unites the members of the family Spirochaetaceae, distinct from the other two spirochaete families. The presence of a laterally acquired gene constitutes a shared characteristic that can be used to identify a particular group, including all the descendants that emerge from the taxon that initially carried the foreign gene [30,31]. Hence, the transferred gene can be used as a unique feature that may reflect the monophyletic nature of the group [30,31], provided the transferred gene is passed on to successive generations either through vertical inheritance or biased HGT.

### Biased gene transfer and the ancient origins of archaeal homeoalleles

Lineages in the living world often are deemed to have arisen solely through a parent-to-offspring genetic transmission, as implied in strictly bifurcating phylogenetic trees that have been largely used in representing evolutionary histories. This concept is mostly applicable to eukaryotic organisms where recombination is linked to procreation through meiosis and sexual reproduction; however, in prokaryotic organisms and even in single-celled eukaryotes, evolution through genetic exchange appears to be more the rule than the exception [32]. In organisms that do not exhibit such reproductive capacities, the tree-like representation may be inaccurate and incomplete because prokaryotes do in fact acquire genetic material through other non-vertical means, such as HGT. The default assumption continues to rest on vertical inheritance as an architect of genealogies. However, some lineages may in fact have been shaped by HGT.

When organisms transfer genetic material with their close kin, the similarity and therefore relatedness observed among them are essentially shaped by gene transfer. We refer to this kind of HGT as biased gene transfer, which involves the acquisition of genetic material from preferred partners based on closer species phylogenetic relationship (i.e. close relatives). This bias is relative to random transfer between any species. HGT bias may also reflect shared ecological niches, susceptibility to similar transfer agents, or symbiotic relationships. In this paper, we limit our discussion to bias between close relatives; however, we note that our definition of HGT biased towards close relatives may lead to circular reinforcement. Organisms that frequently exchange genes become more similar, and transfers between these groups may then be categorized as HGTs with a bias reflecting overall relatedness, even though the initial transfers may have been biased by other factors. We have previously shown that biased gene transfer can create and maintain phylogenetic patterns that resemble the signal created through vertical inheritance [16]. Puigbò *et al*. [33] performed simulations of gene transfer and vertical inheritance that confirmed the possibility of biased gene transfer creating patterns similar to vertical inheritance; although, given their exploration of parameter space, they exclude the possibility that the deep split between Archaea and Bacteria could solely be due to biased gene transfer without any contribution due to vertical inheritance. Unlike transfers that traverse domain boundaries as in the case of the spirochaete PheRS, transfers that occur between close relatives do not exhibit major topological conflicts over large evolutionary distances and this may in fact be initially perceived as a consequence of shared ancestry. However, the occurrence of non-random HGT is evident only at lower taxonomic levels (*e.g*. genus, family), while higher level taxonomic groups (*e.g*. class, phylum) remain cohesive and recognizable [16]. Hence, it may be difficult to ascertain whether the homogeneity of the members of a higher-level group may indeed be a consequence of vertical inheritance or biased gene transfer. In most instances, the two processes will tend to reinforce each other: organisms similar due to recent shared ancestry will remain and become more similar due to biased HGT. Only if HGT is not biased in favor of similarity, as is the case for some extreme thermophilic bacteria [12,13], do shared ancestry and biased HGT create opposing signals.

Studying the distribution patterns of divergent genes with identical functions, referred to as homeoalleles, can reveal preferences for transfer partners [16,34]. Homeoalleles, which are transferred in exchange groups that contains organisms belonging to different higher-level taxa, have been previously shown in phylogenetic analyses of TyrRS wherein two forms of this enzyme exist in Bacteria [16]. Simulations and phylogenetic analyses suggest that biased gene transfer can create and maintain patterns that are indistinguishable from those found in the 16S rRNA phylogeny [16].

**Figure 2:**
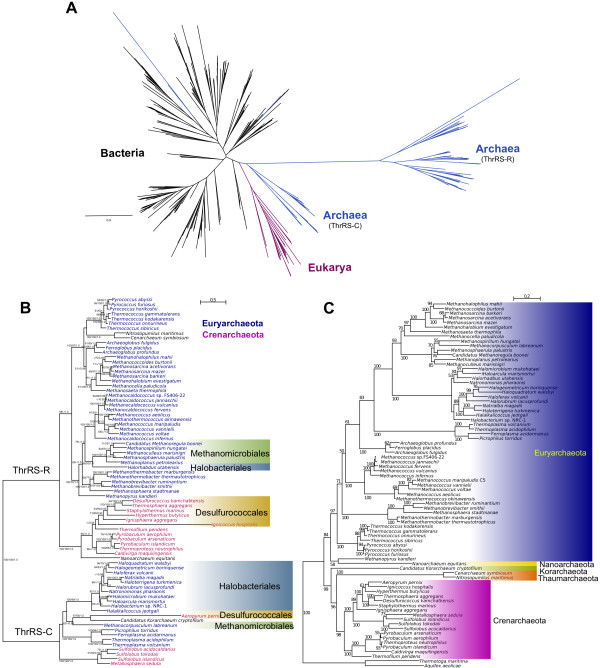
**Phylogenetic analyses of Threonyl-tRNA synthetases (ThrRS)**. (A) Maximum likelihood phylogenetic tree showing the distribution of the two forms of ThrRS across the three domains of life. Branches are color-coded based on the domain. (B) Maximum likelihood phylogenetic tree showing the distribution of the two forms of ThrRS in Archaea, labeled ThrRS-R and ThrRS-C. Taxa names in blue are from the phylum Euryarchaeota and in red are Crenarchaeota. Members of other archaeal phyla are represented in black text. Orders common to both clades are shown in colored boxes. (C) Maximum likelihood phylogenetic tree of Archaea based on concatenated 16S and 23S rRNA sequences. The tree was rooted using bacterial homologs from *Thermotoga maritima *and *Aquifex aeolicae*.

Homeoalleles are also found to exist in the domain Archaea. We generated a maximum likelihood phylogenetic tree based on aligned ThrRS sequences sampled to include representatives from all domains of life. Similar to previous analyses and to the phylogenetic pattern observed in bacterial TyrRS [16,35,36], we detect two types of this enzyme, each forming a well-supported distinct clade (Figure 2A). The more common form of ThrRS, which we refer to as ThrRS-C, is represented in all three domains of life while the rare ThrRS (ThrRS-R) exists only in some archaeal lineages (Figure 2A). Protein sequences show only ~29% identity between the two ThrRS types and it is therefore unlikely that they will undergo homologous recombination. Two pathways for gene replacement are possible. First, HGT can lead to the addition of the novel gene into a genome already possessing a gene of identical function through insertion in another part of the chromosome. Following a period of co-existence, one of the two may eventually be lost. A second mechanism is through homologous recombination of neighboring genes in a syntenic context [16]. Both homeoalleles coexist in higher-level groups (*i.e*., phylum, order), whereas in individual species usually only one of the homeoalleles has been detected. For example, while most members of the Halobacteriales possess ThrRS-C, only *Halorhabdus utahensis *carries ThrRS-R.

The two major archaeal phyla (Euryarchaeota and Crenarchaeota) are represented in both clades, although no single genome carries both forms of the enzyme (Figure 2B). For each clade of archaeal ThrRS, the members of each phyla group together and each phylum remains separate from the other. This is similar to the phylogenetic pattern derived from the 16S-23S rRNA sequences showing the two major archaeal phyla as monophyletic (Figure 2C). For taxonomic categories lower than the phylum level, the orders Methanomicrobiales, Halobacteriales and Desulfurococcales, are found to be represented in both ThrRS clades and consist of members that carry one or the other type of ThrRS.

We previously found that two forms of SerRS that behave as homeoalleles exist in Euryarchaeota [34]. Similar to the patterns in archaeal ThrRS, we also observed two divergent forms of SerRS in Archaea, with one form present in the three domains of life (SerRS-C) and a rare form present only in a few members of Archaea (SerRS-R). The rare form has only been found in 18 genera and 5 orders belonging to the phylum Euryarchaeota. In even rarer cases we find a genome carrying both SerRS forms, as in *Methanosarcina barkeri*, and this occurrence may reflect a period of transition in which one SerRS is in the process of replacing the other. This implies that individual lineages can acquire different versions of a homeoallele through HGT, and they can also be lost from the lineage and replaced by another version.

**Figure 3:**
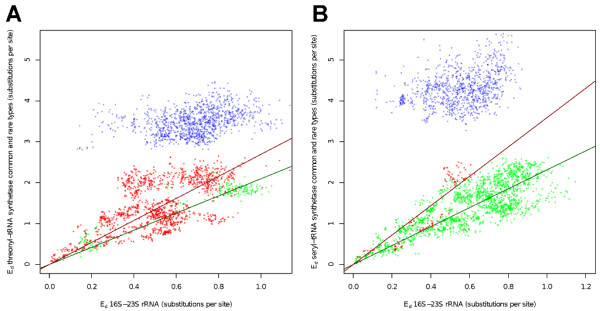
**Scatterplots of pairwise evolutionary distances between the combined 16S-23S rRNA (x-axis) and (A) ThrRS and (B) SerRS sequences on y-axis from a diverse sampling of bacteria**. In (A), distances between taxa within the ThrRS-R clade (plotted in red) show a strong correlation with the rRNA distances (R^2 ^= 0.90, P < 2.2 × 10^-16^), as do the distances within the ThrRS-C plotted in green (R^2 ^= 0.98, P < 2.2 × 10^-16^). Pairwise distances for *Aeropyrum pernix *and *Ignicoccus *hospitalis were not included in the scatterplot because of the high rate of sequence divergence (long branches in Figure 2B). A strong correlation between the pairwise distances is expected for two trees with similar topologies. In (B), the distances for each clade show a strong correlation with the rRNA distances. Distances with the SerRS-R are plotted in red (R^2 ^= 0.94, P < 2.2 × 10^-16^) and SerRS-C in green (R^2 ^= 0.95, P < 2.2 × 10^-16^). Pairwise distances between each clade are shown in blue.

To assess the agreement in phylogeny for each form of ThrRS and SerRS with the rRNA, we plotted the pairwise distances in ThrRS sequences against their corresponding distances in 16S-23S rRNA for each pair of taxa (Figure 3A). These types of plots have been previously used to detect gene transfers [36,37]. However, in this study, we did not use this approach to show HGT but to illustrate the correlation between each of the two aaRS clades and the rRNA tree. Each ThrRS clade exhibits a phylogenetic pattern similar to the rRNA phylogeny represented by the strong correlation in the distances between each pair of organisms, indicating similarity between the phylogenies of each ThrRS clade with the expected ribosomal tree (Figure 3A). We show that a positive correlation exists for each clade, despite the occurrence of gene transfer, and that there is no obvious phylogenetic trace of HGT that can be detected by phylogenetic conflict within each clade, because the transfers occur between taxa with close phylogenetic affinity. Within each ThrRS type, the distances between pairs of ThrRS homologs and the corresponding rRNA gene pairs show a strong correlation (Figure 3A) [For pairwise distances between rRNA and (A) ThrRS-R R^2 ^= 0.90, P < 2.2 × 10^-16^, ThrRS-C R^2 ^= 0.98, P < 2.2 × 10^-16^. R^2 ^is the square of the correlation coefficient]. These results indicate that within each of the two ThrRS clades, evolution is similar to the expected ribosomal phylogeny, but if we consider the ThrRS phylogeny as a whole, we observe a conflicting signal. The distances between the two ThrRS types do not correlate to the corresponding 16S-23S rRNA distances (in blue), but rather reflect the ancient divergence between the two ThrRS types. We obtained similar results for the SerRS data, with the distances between pairs of SerRS homologs and the corresponding rRNA gene pairs showing a strong correlation (Figure 3B) [SerRS-R R^2 ^= 0.94, P < 2.2 × 10^-16^, and SerRS-C R^2 ^= 0.95, P < 2.2 × 10^-16^].

**Figure 4:**
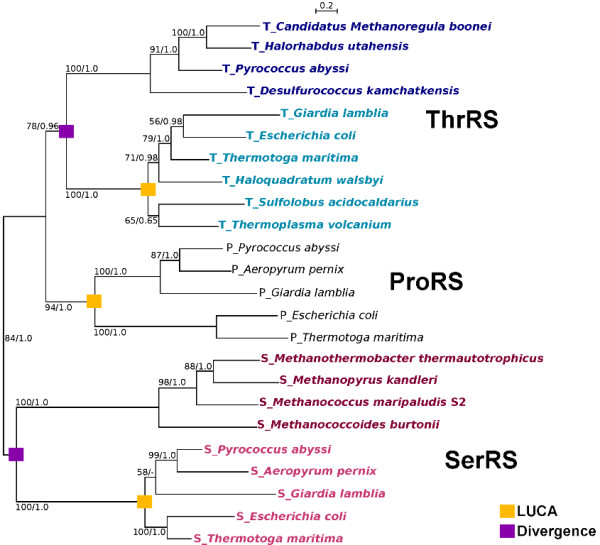
**Phylogenetic analyses of representative Class II aaRS showing the divergence of the two forms of ThrRS and SerRS**. Taxa that contain the rare form of the enzymes ThrRS and SerRS are represented in dark blue and red, respectively. Those that possess the common form (*i.e*. they are found across the three domains of life) are represented in light blue and pink.

Two distinct, unrelated classes of aaRS exist (I and II), with each class defined by unique domain structures and sequence homologies. Both ThrRS and SerRS are members of the Class II aaRS [38,39]. To account for the rare occurrence and dispersed distribution of the rare forms of SerRS and ThrRS, we propose that the rare forms of these two enzymes diverged early from the common forms, with subsequent horizontal transfer from an unknown ancient lineage that probably has gone extinct or is yet to be discovered. Phylogenetic reconstruction based on representatives from several Class II aaRS shows that an ancient divergence gave rise to the common and rare forms of both ThrRS and SerRS (Figure 4). Indeed, this divergence event appears to have occurred prior to the Last Universal Common Ancestor (LUCA) node of SerRS-C and ThrRS-C (defined as the common ancestor of Bacteria and Archaea homologs; Figure 4). This implies that many other lineages may have co-existed with LUCA and have contributed to the genetic composition of LUCA's descendants [40]. The early branching lineages may have also acquired these ancient genes and have persisted in present-day genomes. One example is pyrrolysyl-tRNA synthetase (PylRS) that emerged in the pre-LUCA era and is now only found in a few members of Bacteria and Archaea [41]. The phylogenetic distribution of this enzyme suggests that these extant taxa acquired PylRS through several HGT episodes from an ancient, most likely extinct, lineage [41]. Indeed, this finding provides evidence that the most recent common organismal ancestor and the molecular most recent common ancestors (*i.e*. ancestors of a particular gene) may not have coincided in time and space [40] and ancient genes from long-extinct organisms may continue to survive in modern-day organisms through HGT.

The archaeal homeoalleles provide additional evidence, in addition to bacterial TyrRS [16], that biased gene transfer can indeed re-create phylogenetic patterns that resemble those generated through common ancestry. Hence, the organismal lineages of Archaea and Bacteria reflect a combination of vertical inheritance and biased HGT, and may be best represented by a line of descent that has access to a gene pool containing genetic material, including homeoalleles, from various sources. This gene pool, or mobilome [42], is not limited to the genome of a particular organism; genomes of closely related taxa, as well as mobile genetic elements (phages, transposons, plasmids) [42], also act as sources for DNA that an organism can exploit. What we see are lineages that exhibit continuity from beginning to end as a consequence of vertical inheritance and biased gene transfer, with genes moving in and out of the lineage through time. In some instances, as in the case of the inter-domain transfer of PheRS, or the extreme thermophilic bacteria, horizontally acquired genes may come from more evolutionary distant sources, more as a consequence of shared ecological niches [13] and may indeed create highways of gene transfer between distant relatives [11].

The more genes that two lineages transfer between each other, the more similar that the lineages become and the more frequently that they will continue to exchange genes [16]. If genes are mainly transferred between close kin, then gene transfer reinforces similarity, regardless if it is because of shared ancestry or biased HGT [16]. More frequently, biased gene transfer will reinforce similarity due to recent shared ancestry. Thus, instead of eroding the phylogenetic signal retained in a genome, HGT may act to reinforce groups created through vertical descent [16,43]. The challenge remains on how to clearly distinguish between patterns created through vertical inheritance and through biased transfer. In the case of homeoalleles, one can infer gene transfer from the distribution of the different homeoalleles, provided one cans use a reference tree that approximates shared ancestry, such as a tree based on all the components of the ribosome. The LGT3State approach [44] was successfully used in [16] and [45] to estimate the transition probabilities of two divergent enzymes under either gene loss only or HGT/loss scenario using maximum likelihood. For both TyrRS and SerRS the analyses showed that its distribution in extant lineages can be better explained through the HGT and loss model than through gene loss alone [16]. However, to differentiate clearly patterns created by vertical inheritance and biased HGT in the absence of homeoalleles will require further data and analyses.

As a result of biased transfer, we expect to observe similar distribution patterns between a specific gene tree and the ribosomal tree. While biased HGT can give rise to phylogenetic patterns similar to those created through shared ancestry, both processes occur simultaneously in nature. Through biased transfers, a group then may be defined by the multiple transfers of the same gene among different closely related taxa, generating cohesion among the recipient organisms in terms of genetic similarity.

## Conclusions

Graham Cairns-Smith introduced the metaphor of a rope to illustrate a line of decent, with the long overlapping fibers representing individual gene histories [46]. To illustrate the continuity of a lineage during a genetic takeover, he considered a rope consisting of hemp fibers at one end that is gradually transformed into a rope with only sisal fibers present, with hemp fibers gradually fading out and sisal fibers fading in. Gary Olsen (University of Illinois, Urbana-Champaign) used this metaphor to argue for the continuity of a lineage in prokaryotic evolution even in the presence of rampant HGT [31]. The image of a rope has a long history to illustrate continuity in the presence of turnover and change (e.g., [47]). The rope (*i.e*. lineage) itself is continuous and not reticulated, but the lineage is embedded in a reticulated genome phylogeny and composed of a mèlange of genetic materials that have come and gone over time [31]. In a similar fashion, the Tree of Cell Devisions (ToCD) based on the genealogy of cells has recently been proposed as a tree-like process representing an important aspect of the history of life ([48-50], see Ford Doolittle's review in [51]). The concept of the ToCD may reflect an important aspect of organismal evolution, and most genes and cellular functions remain vertically inherited over short time spans of only a few generations. Thus, the ToCD may be well-defined for some groups of organisms, just as the rope metaphor allows an organismal lineage to be defined even in the presence of rampant HGT; however, the reconstruction of the ToCD has to be based on inherited characters, and for these it remains uncertain whether they were acquired through biased HGT or vertical inheritance.

The evolutionary history of prokaryotes is convoluted largely as a result of microorganismal abilities to accept foreign genes and incorporate them into their genomes. For ancient transfers, it becomes more problematic to trace the history of the transferred gene in part because the donor organism's lineage might have gone extinct since the transfer took place. Relationships and lineages in the microbial world are shaped by both HGT and shared ancestry that, over long periods of time, will exhibit a continuous genealogy. In most instances, transfer bias will maintain and strengthen similarity within groups. Only in cases where HGT bias is due to other factors, such as shared ecological niche [12,13], do patterns emerge from gene phylogenies that are different from the ToCD.

## Methods

Protein sequences of PheRS, ThrRS, SerRS and ProRS from Archaea, Bacteria and Eukarya were retrieved by BLASTP searches of the non-redundant protein database and BLAST microbial genome database from the National Center for Biotechnology Information (NCBI) website [52]. Sequences were aligned using the MUSCLE algorithm [53] with default parameters. Maximum likelihood phylogenetic reconstruction of the aaRS sequences was performed using PhyML v3.0 [54] with 100 bootstrap replicates, WAG [55] substitution model, estimated portions of invariable sites, four substitution-rate categories, estimated Γ distribution parameter, estimated amino acid frequencies, and 20 random starting tree. Distances between sequences were calculated using the programs PUZZLEBOOT v1.03 [56] and TREE-PUZZLE [57] using the WAG [55] +Γ+I model to calculate pairwise maximum likelihood distances and NEIGHBOR [58] for tree reconstruction. Posterior probability values were generated using MrBayes v. 3.1.2 [59], with a fixed WAG [55] amino acid substitution model using four rate categories approximating a Γ distribution, four chains, a swap frequency of 1, and a random starting tree. We used a specified number of generations for each aaRS analyses (200,000 for the small PheRS α subunit, 130,000 for small PheRS β subunit, 650,000 for archaeal ThrRS and 800,000 for archaeal SerRS, sampling every 100th generation. The first 25% of the sampled generations were removed from the analysis as burn-in. Nucleotide sequences for the 16S rRNA and 23S rRNA were obtained by BLASTN searches and their alignments were concatenated. For the 16S-23S rRNA phylogeny, tree reconstruction and bootstrapping were performed using PhyML v3.0 [54] with estimated portions of invariable sites, four substitution-rate categories, estimated ts/tv ratio, estimated Γ distribution parameter, estimated amino acid frequencies, BioNJ starting tree, 100 bootstrap replicates and GTR [60] nucleotide substitution model. Branch lengths and topologies of all phylograms were calculated with PhyML v3.0 [54]. The substitution models used for each approach were determined using ProtTest [61] and jModelTest [62]. Pairwise distances between sequences were calculated using TREE-PUZZLE [57] using the WAG [55] +Γ+I model.

## Abbreviations

aaRS: aminoacyl-tRNA synthetases; HGT: horizontal gene transfer; LUCA: Last Universal Common Ancestor, PheRS: Phenylalanyl-tRNA synthetase, PylRS: pyrrolysyl-tRNA synthetase, SerRS: Seryl-tRNA synthetases, ThrRS: Threonyl-tRNA synthetases; ToCD: Tree of Cell Divisions

## Competing interests

The authors declare that they have no competing interests.

## Authors' contributions

JPG conceived of the study. JPG and CPA participated in its design, data analyses and wrote the manuscript. All authors read and approved the final manuscript.

## Reviewers' comments

### Reviewer 1

W. Ford Doolittle, Department of Biochemistry and Molecular Biology, Dalhousie University.

In this interesting and eclectic paper, Cheryl Andam and Peter Gogarten present a phylogenetic analysis of several aminoacyl-tRNA synthetases, drawing some general conclusions about LGT which, if not altogether new, remain important.

First, they show quite convincingly that the PheRS of three spirochaete genera represent a single transfer from archaea, prior to their divergence, and that both donor and recipient were likely to have been thermophiles. Less convincingly they propose that a more heat-stable PheRS would have helped the recipient "satisfy the increased demand for Phe incorporation into proteins to provide additional stability to their proteins." Maybe, but some actual thermostability measurements on the existing enzymes and better still on their reconstructed common ancestor are clearly called for-as well as some idea of just how much the Phe content of the thermoadapting lineage would have had to increase. More convincingly, Andam and Gogarten point out just how nicely such rare transfers serve as synapomorphies.

*Authors' response: We thank the reviewer for his comments and suggestions, and we agree that measuring the thermostability of existing enzymes and the reconstructed ancestor is an essential next step. However, we think that this would be more appropriate for a separate study in the future*.

*WF Doolittle's review continued: *Second, the authors expand evidence in support of an idea I first heard Prof. Gogarten articulate maybe 15 years go at a meeting in Banff, and have liked ever since. This is that we expect, for a great many reasons, that (1) lateral transfers will decrease in likelihood (of happening and of being fixed) the greater the phylogenetic distance between donor and recipient, and thus (2) LGT is more likely to preserve than destroy any Tree of Life. LGT will to be sure create a certain degree of fuzziness, but at a distance one won't see it.

This has always been a bit difficult to prove, though, because the closer the donor and recipient the harder it will be to detect transfers, just from the sequences of their genes. So what Andam and Gogarten do here, in an extension of their recent PNAS paper, is address an interesting situation, in which there appear to be two distinct homeoallelic versions of a synthetase, each giving a close approximation to the expected (rRNA) tree but mixed in together, so that sometimes it will be one and sometimes the other that places a species where it "belongs". There are two ways to explain this. First, supposing that all genomes have had both alleles until very recently and then they in some sort of mysterious genomic equivalent of quantum entanglement have lost one or the other, more or less at random. Or second, that both forms have been "kicking around", like alleles but at a higher than species level, and are being substituted for each other by LGT ("non-orthologous replacement") at a goodly rate, but only over short evolutionary distance. This would make the Tree of Life look fuzzy up close but not change it at a distance (just as incomplete sorting of alleles shortly after speciation or a limited amount of within-genus hybridization will not confuse relationships between major groups of animals, say).

*Authors' response: We agree with the reviewer that, at the larger scheme of life's history, biased gene transfer does make the branches of ToL appear fuzzy but its overall structure or pattern remains the same, i.e. major prokaryotic groups remain recognizable. Also, it may be worth reiterating that the biased gene transfer we describe is *not necessarily *rampant, and that vertical inheritance does make a contribution to the similarity of phylogenies calculated for different molecules*.

*WF Doolittle's review continued: *These authors' recently published PNAS paper applied this reasoning to bacterial TyrRS: here they have focused on archaeal ThrRS and SerRS, each of which has a common and a rare form. With the former they show that a "positive correlation [with rRNA phylogeny] exists for each clade, despite the occurrence of gene transfer, and that there is no obvious phylogenetic trace of HGT that can be detected by phylogenetic conflict within each clade, because the transfers occur between taxa with close phylogenetic identity."

It's hard to think up a reasonable alternative explanation, though these results do make one wonder, first why it is that the probable intermediate state (two synthetases in the same genome) is so seldom observed, and second what it is that biases LGT so strongly. If it is co-evolved fits between the tRNAs and the synthetases, there is some obvious old-fashioned mix-and-match biochemistry that could easily be done.

*Authors' response: The rare co-existence of divergent enzymes with identical function remains puzzling. Some duplicate aaRS in bacteria confer differential antibiotic resistance against natural competitors *[63]. *And this might be true as well for the Archaea (archaeal antibiotics halocins and sulfolobicins, collectively called archaeocins; *[64]). *One possible explanation is that there is a relatively quick transition/switch between the two types as a result of a stochastic loss of one or the other. This could be due to the antibiotics targeting one of the homeoalleles being present only for short periods of time, and in the absence the duplicate aaRS, becomes redundant. Another finding that may contribute to the rare co-occurrence of the two homeoalleles is that the replacement might not always occur via the intermediate state of the two different homeoalleles coexisting *[16]. *Rather, the switch can be accomplished through homologous recombination in the flanking regions. In agreement with the latter scenario, the two different tyrRS homeoalleles in Gamma- and Deltaproteobacteria are found in the same syntenic context in different genomes*.

*At present we do not have a strong opinion on which of the many possible factors biases the transfer in favor of close relatives. However, we do not think that the co-evolution with tRNAs makes a major contribution, because for some tRNAs different types are found in bacteria and archaea, but the distribution of these types does not correspond neither to the ribosomal phylogeny or to the distribution of aaRS homeoalleles, suggesting that both, tRNA and aaRS are transferred independently (Andam, Papke, Gogarten, manuscript in preparation)*.

*A more heretical explanation for the prevalence of gene transfer between organisms that we consider related is that we find this bias, because it is the main driving force in creating similarity between organisms, and for creating shared ancestry in the genes of organism that we consider related. If biased transfer is the main force in defining groups at higher taxonomic level, then it is not surprising that most of the transfers are within this group. The power of biased gene transfer to create overwhelming phylogenetic signal is illustrated by the genomes of the Thermotogae and Aquificae *[12,13]: *If their ribosomes reflect vertical inheritance, then these organisms have traded nearly all their other genes with Clostridia, Archaea and Epsilonproteobacteria, respectively, to the extent that one may consider them as Clostridia (in case of Thermotogae) and Epsilonproteobacteria (in case of Aquificae) that picked up their ribosomes from a deep branching bacterial lineage*.

*WF Doolittle's review continued: *Andam and Gogarten also propose that the common and rare forms of Thr and SerRS diverged prior to the time of LUCA. If I understand the reasoning here, it is formally related to that used by Gogarten and colleagues 22 years ago to root the universal tree. Then, paralogous gene families represented in all three domains were used to root the universal tree on the assumption that the duplication producing them was pre-LUCAn. This assumption looked at that time almost unchallengeable because each of the paralogs produced the same tree (rooted by the duplication). It would have seemed perverse in 1989 to suppose the actually what had happened was (1) "horizontal transfer from an unknown ancient lineage that probably has gone extinct or is undiscovered" followed by (2) frequent but biased HGT, so that the same "correct" three-domain tree is recreated. But this is exactly what is proposed here to explain aminoacyl RS phylogenies. So it seems to me that the notion of biased gene transfer, true though it may be, deconstructs much of what has gone before. I am looking forward to Andam and Gogarten explaining to me how I have got this wrong.

*Authors' response: We agree with the reviewer that gene transfer makes rooting the tree or net of life more difficult. With the recognition that gene transfer occurs frequently, we can no longer assume that the rooting of the ATPsynthase or elongation factor phylogenies also pertains to the ribosomal phylogeny. However, the rooting between the bacterial and archaeal domains is recovered for many molecular systems (most, if one only considers the ones with sufficient phylogenetic information, and if one allows for occasional transfers between the domains). The same placement of the root is also recovered for the ribosomal proteins, when one uses the echo from the assembly of the genetic code to determine the most ancient branch *[65]. *Thus we regard the split between Bacteria and Archaea as a fundamental divergence, reflected in many molecular phylogenies. While this split is fundamental, we do not think that all genes trace their lineage back to a single organism that harbored all the most recent common ancestors (molecular LUCAs). Rather a population distributed in space and time carried the molecular LUCAs. (We are still fond of the analogy to the human population *[40]-*there was a mitochondrial Eve and a Y chromosome Adam, but about 10,000 other humans also contributed genes to modern humans.)*

*Gene transfer makes the discussion of LUCA more complicated, but in our opinion the situation is not hopeless. In case of SerRS*, sensu strictu *the molecular LUCA has to be placed at the divergence of the rare and common form of the enzyme. However, in contrast to the ATPsynthases and elongation factors, one of the genes that evolved from the ancestral divergence does not have universal distribution. The disjunctive distribution of the rare SerRS from suggests its transfer between methanogens after it was first acquired from the deep branching lineage. Nevertheless, the reviewer is correct in suggesting that one could use the rare forms as an outgroup for a phylogeny of the common form. Given the possibility of long branch attraction, and the limited amount of phylogenetic information retained in a single molecule, the results have to be interpreted with caution; however, the rare form of SerRS places the root on the bacterial branch of the phylogeny of the common form*.

*WF Doolittle's review continued: *Indeed it seems to me that once one (1) accepts as a logically unassailable necessity that many and perhaps all of the last common ancestral *organism*'s genes have been replaced by genes whose ancestors were at that time present in the genomes of contemporaneous organismal lineages that (by definition) have since gone extinct, and (2) admits as a reasonable possibility that biased transfer can replace vertical descent as the force which maintains-but may sometimes rearrange-the branches of the universal tree, then all bets are off on making any firm conclusions about really deep phylogeny, conclusions of the sort Andam and Gogarten wish to make here.

*Authors' response: We think the reviewer is overly pessimistic. Most transfers are between close relatives, thus the overall shape of molecular phylogenies still resembles organismal history. Yes, given any genome, every single gene over billions of years has likely been replaced by a transferred gene. But most of the genes were acquired from close relatives, thus the overall shape of the tree remains the same. In addition, many cases of gene transfer between domains and between phyla can be detected from comparisons between different gene phylogenies. There are cases where it may be difficult or impossible to decide between different alternative explanation, such as the distinction between ancient divergence followed by differential gene loss and gene transfer. The identification of the organismal lines of descent in the tangle of gene phylogenies is difficult, not entirely accurate, and for some organisms may even be impossible; however, we remain optimistic that molecular phylogenies hold the key to determining the often reticulated phylogenies of extant groups of organisms. Furthermore, because of gene divergences that occurred before the organismal LUCA, molecular phylogenies also shed light on the pre-LUCA evolution of information processing and metabolism*.

### Reviewer's report 2

François-Joseph Lapointe, Département de Sciences biologiques, Université de Montréal.

I read with great interest this paper and found it acceptable for publication in Biology Direct. I agree with the conclusion of the authors about biased HGT and its effect on sequence similarity. However, I believe that using the same terminology for different processes can be misleading. Sequence similarity is not the same as genealogy. The processes of vertical inheritance and biased horizontal gene transfer are quite different things, regardless of the outcome. It is of interest to detect similarities in patterns, but also to detect discrepancies in processes. I would like to see the authors focus not only on biased HGT, which mimics genealogy, but on proposing ways to discriminate both types of gene transfer.

*Authors' response: We agree with the reviewer that these two processes are undeniably different, however, as we have discussed elsewhere *[16], *the patterns generated by each process may be indistinguishable. Unfortunately, at present our conclusion is that to some extent phylogenomics may result in a phenetic classification *[66], *but the extent of this problem is at present unknown*.

*FJ Lapointe's review continued*: Here are some specific comments:

1) A formal definition of "biased HGT" is not provided, and can mean a lot of different things. I can think of four (at least) possible interpretation of the term.

i) A directional bias (A-> B is not as likely as B-> A).

ii)A functional bias (genes with the same function are more likely to be transferred)

iii) A taxonomical bias (some taxa-related or not-are more prone to HGT)

iv) An environmental bias (taxa living in the same environment are more likely to exchange genes with one another)

The authors need to be precise about what they mean by "biased HGT". Depending on their use, different processes are involved and detection of biased HGT may be more or less difficult.

*Authors' response: We apologize for the oversight and have included a definition of the term "biased gene transfer" in the manuscript*.

*FJ Lapointe's review continued*: 2) It is not clear to me how biased HGT can be easily detected, and not mistaken for ancient gene duplication. Of course, one can detect gene duplication when multiple copies of a gene are still occurring in a given taxon, but this is not always the case. In such situations, how can one distinguish duplication from HGT? In Figure 4, divergence can either mean HGT or gene duplication.

*Authors' response: We would like to make two distinct sets of comments: The first concerns the difficulty of distinguishing between ancient gene duplication followed by differential gene loss and recent gene transfer. In principle, any molecular phylogeny that disagrees with the organismal or reference phylogeny can be explained by either process *[7]. *The decision about which explanation to favor depends on the frequency of convergent gene-loss events. In case of the distribution of homeoalleles, we used the LGT3State model *[44]*to ascertain that scenarios with gene transfer are more probable than a gene-loss only model, given the data *[16,34].

*Second, what traditionally is considered a gene duplication event, in many instances upon closer inspection will reveal itself to be the result of divergence followed by gene transfer and non-homologous recombination of the divergent copy *[66,67]. *Given that autochthonous gene duplication in a lineage is a rare occurrence, we prefer the use of the term divergence, to cover both HGT and duplication. Analysis of the deep divergences between the aaRSs with different amino acid specificity may inform us on the expansion of the genetic code, but this is beyond the scope of the current manuscript*.

*FJ Lapointe's review continued*: 3) The authors have a long series of papers, using both empirical data and simulations, which show the similarities between monophyletic clades as a result of vertical inheritance, and the clades as a result of biased HGT. I am not disputing the fact that shared derived characters can emerge in both cases. Still, one can argue that these so-called "synapomorphies" are not produced by the same processes; thus, they should not be called the same. It is important to define a new vocabulary to characterize these groups, not simply to co-opt cladistic terminology. Otherwise, the same old words (monophyly, synapomorphy, clade) pertaining to tree-like evolution will still be used to describe totally different processes (and we know that some of these processes cannot be represented by a tree). This can be quite confusing.

*Authors' response: Again, we would like to give a two-part answer: First, if a gene acquired through HGT is inherited through vertical descent by all descendants of the recipient, then the presence of the transferred gene is indeed a synapomorphy in the cladistic sense *[68,69]. *There is no reason to invent a new term for this case. In reality things can be complicated through lineage sorting and gene loss *[29], *but in principle the cladistic approach appears valid. Second, the case of biased gene transfer distributing a gene within a group is different, as exemplified in the rare form of SerRS. So far, this homeoallele has only been found in methanogens, but its distribution within the methanogenic archaea suggests spread not only by vertical inheritance, but also through biased HGT. The frequent occurrence of gene transfer suggests caution in interpreting the presence of a transferred gene as a synapomorphy. The interpretation of an atypical gene as a synapomorphy has to be regarded as a hypothesis, and especially in case of limited taxon sampling may be falsified by future findings. Cladistic and phenetic similarities can indeed be difficult to differentiate, and if apparent synapomorphies are frequently revealed to be the consequence of biased transfer, then a new terminology for the latter might be useful to describe the phenetic group defined by gene transfer bias*.

*FJ Lapointe's review continued*: 4) In Figure 3, pairwise evolutionary distances are compared, but details of the computations are not provided. Are these pairwise distances among sequences, corrected distances or path-length distances in the corresponding trees?

*Authors' response: We apologize for the oversight and we now included a statement on how the pairwise distances were calculated*.

### Reviewer's report 3

Frédéric Bouchard, Department of Chemistry-Biochemistry and Centre Pharmaqam, Université du Québec à Montréal.

I will focus my comments on some of the broad theoretical dividends of the paper. Because of this, I may be overreaching in my interpretation of the findings of this paper. Regardless, the paper is highly stimulating and deserves broad readership. It deserves publication with minor precisions.

The paper highlights two results.

1- that vertical processes and horizontal processes may lead to similar patterns

2- that horizontal processes may in fact reinforce the appearance of verticality because of biased HGT

The second point is not as explicit but in my mind is the most novel point. Some claim that horizontal transfer may seem invisible because: a) HGT is infrequent in some/most lineages; b) it occurred too far in the past; c) it 'merely' replaced something that was similar or closely related. This could explain why vertical patterns and vertical+horizontal patterns would be similar. As the authors point out, the three aforementioned explanations are not as compelling as previously thought because of the now recognized pervasiveness of HGT. This makes their second proposition that biased HGT may reinforce similarity between closely related organisms all the more intriguing. If they are correct, then there would be a virtuous circle between vertical processes and horizontal processes in that organisms that are closely related by vertical inheritance would exchange genes more readily thereby increasing their genetic and functional (?) similarity. This may mean ironically that HGT could explain why vertical patterns seem so cohesive and exhaustive! It would be BECAUSE of biased HGT that vertical processes appear sufficient to explain the patterns we observe.

If that is correct, then the author may want to go for a bolder claim (maybe in future research projects): the patterns would be similar (undistinguishable) only if biased HGT is weak. In case of strong biased HGT, we should expect patterns created by vertical processes alone to be "looser" (less defined) than ones created by horizontal and vertical processes i.e. the patterns would not be undistinguishable.

*Authors' response: We thank the reviewer for his comments and the suggestion. One way to identify if biased transfer led to the cohesion of a prokaryotic group is that within-group phylogenetic patterns should reveal conflicts *[15], *whereas between-group transfer do not. In case of homeoalleles the transfer is inferred from the distribution of the divergent homeoallele types, and for each clade of homeoalleles the phylogenetic patterns indeed do not match the ribosomal phylogeny perfectly *[16]. *However, these conflicts provide only corroborating evidence, because it often is difficult to distinguish conflicts due to a lack of phylogenetic information from conflicts due to within-group transfer. The reviewer is correct that in case a group is created and held together through biased gene transfer, the transferred genes should result in tighter, more recent divergence of the group than genes that were vertically inherited. Two problems in translating this idea into a testable hypotheses are as follows: First, it is difficult *a priori *to decide which genes are vertically inherited and which were transferred. Homeoalleles provide a unique opportunity to identify reliably within-group transfer from the distribution of the allele types. It seems reasonable to assume that the transfer rates in case of other genes are as high; however, in the absence of homeoalleles the detection of these transfers can only be based on within-group conflicts of molecular phylogenies, thus the identification of transfers is less decisive. Second, the tightness of the group would be reflected in more recent apparent shared ancestry; however, molecular phylogenies are usually scaled with respect to substitutions and the inference of substitution rates is often problematic. Only if large sets of genes are analyzed in a comparative study could one hope to detect the tighter grouping that results from frequent biased transfer*.

*F Bouchard's review continued*: Maybe I misunderstood the goal of the paper or the strength of the evidence because of the relative lack of definition of the key concept of "biased HGT". I wasn't certain what the authors meant by that expression. The usage of the expression in the paper clearly refers to HGT between closely genealogical organisms, but the mention of shared ecological niches suggests that it may be between similarly functioning organisms (in addition or sometimes independently of their shared history). The word "species" is often jettisoned in these discussions because of the theoretical baggage, but here it seems we are coming back to the same issues. Lineage is usually a historical concept (i.e. a multi-generational succession of related organisms, or something similar), which matches many intuitions we have concerning species. But in the debate about the definition of species, some have adopted a purely ahistorical functional concept of species, such as Van Valen's Ecological Species Concept. This text seems to adopt a hybrid notion of lineage one that comprises organisms that are genealogically related AND operating in similar/shared niches. Could biased HGT occur between genealogically related organisms that operate in distinct niches? Would that count as biased HGT or just HGT? A more detailed definition of biased HGT would help answer this question and would provide a better idea of how they wish to redefine the notion of lineage. But maybe this is for future research projects.

*Authors' response: We apologize for not being specific about our definition of biased HGT. We added a discussion of the definition of biased gene transfer and the type of bias we discuss in the manuscript. We focus our analyses on transfers between closely related organisms. Transfer bias can be created through different processes, such as shared ecological niches, symbiotic relationships, susceptibility to transfer agents, similar genome organizational signals, nucleotide and codon usage biases, similarity in regulatory sequences, etc.. Many of these factors will bias transfer in favor of close relatives, but highways of gene sharing were described between unrelated organisms *[11,70]. *However, in this manuscript we limit our discussion to bias in favor of transfer between close relatives*.

*F Bouchard's review continued*: In addition to a more explicit definition of biased HGT, let me add a minor point where I think the argument could be better presented

"When organisms transfer genetic material with their close kin, the similarity and therefore relatedness observed among them are essentially shaped by gene transfer" (p.6) The bold (not pejorative here!) claims this section makes hinge on references [16] and [33], yet the authors claim that [16] shows the possibility of pattern similarity. Possibility is different from reality... and therefore a broader discussion of [16] would help bolster the claims made in this section. The whole section aims to do this, but I would offer a longer discussion/analysis of [16] in this paper.

More generally I would have liked a slightly longer discussion of how to distinguish patterns that could have emerged purely via vertical inheritance and patterns that could have emerged via vertical inheritance and biased HGT. Since the argument here is that it is possible for vertical patterns to have emerged via vertical+HGT processes, it would be desirable to make more explicit why the latter more complicated story is to be favoured contra a more parsimonious (but wrong) story of simple trees with simple branches. The argument aims to do this, but by repeating that the patterns are undistinguishable, the authors rely too strongly on parsimony alone, I think. But parsimony is a double edged sword: the "opponents" of HGT invoke parsimony as well. The authors need to explain why their parsimony is more parsimonious...

*Authors' response: We agree, there is a big difference between possibility and showing that things actually happened. Our hypothesis that biased HGT may create patterns usually mistaken as due to shared ancestry began with a thought experiment *[71], *i.e., showing the possibility, but not that this actually had happened in nature. As discussed above, corroborating this hypothesis was difficult, because the predictions from this hypothesis with respect to molecular phylogenies relied on phylogenetic conflicts that often were too small to be convincing. This situation changed with the discovery of homeoalleles in bacteria and archaea *[16,34]. *As is true for all gene transfer events, the replacement of one homeoallele with another can in principle be explained by an ancient gene duplication followed by convergent gene loss events. However, the likelihood for the loss-only model was found to be significantly smaller (see the discussion of the LGT3State model above). Furthermore, the finding that some of the replacements between divergent homeoalleles occurred though recombination in the more conserved flanking regions, maintaining a syntenic gene neighbourhood for the divergent homeoallele types *[16], *shows that at least in these cases the two homeoalleles did not previously coexist in the same genome*.

## References

[B1] Jain R Rivera MC Moore JE Lake JA Horizontal gene transfer accelerates genome innovation and evolution Mol Biol Evol 2003 20 10 1598 1602 10.1093/molbev/msg154 12777514

[B2] Hehemann JH Correc G Barbeyron T Helbert W Czjzek M Michel G Transfer of carbohydrate-active enzymes from marine bacteria to Japanese gut microbiota Nature 2010 464 7290 908 912 10.1038/nature08937 20376150

[B3] Caro-Quintero A Deng J Auchtung J Brettar I Hofle MG Klappenbach J Konstantinidis KT Unprecedented levels of horizontal gene transfer among spatially co-occurring Shewanella bacteria from the Baltic Sea ISME J 2010 in press 10.1038/ismej.2010.93 PMC3105679 20596068

[B4] Martiny AC Huang Y Li W Occurrence of phosphate acquisition genes in Prochlorococcus cells from different ocean regions Environ Microbiol 2009 11 6 1340 1347 10.1111/j.1462-2920.2009.01860.x 19187282

[B5] Treangen TJ Rocha EPC Horizontal Transfer, Not Duplication, Drives the Expansion of Protein Families in Prokaryotes PLoS Genet 2011 7 1 e1001284 10.1371/journal.pgen.1001284 21298028 PMC3029252

[B6] Williams D Andam CP Gogarten JP Oren A, Papke RT Horizontal Gene Transfer and the Formation of Groups of Microorganisms Molecular Phylogeny of Microorganisms 2010 Caister Academic Press http://gogarten.uconn.edu/articles/WilliamsAndamGogarten_HGT_and_the_Formation_of_Groups_of_Microorganisms.pdf

[B7] Gogarten JP Townsend JP Horizontal gene transfer, genome innovation and evolution Nat Rev Microbiol 2005 3 9 679 687 10.1038/nrmicro1204 16138096

[B8] Kloesges T Papa O Martin W Dagan T Networks of gene sharing among 329 proteobacterial genomes reveal differences in lateral gene transfer frequency at different phylogenetic depths Mol Biol Evol 2011 28 1057 1074 10.1093/molbev/msq297 21059789 PMC3021791

[B9] McDaniel LD Young E Delaney J Ruhnau F Ritchie KB Paul JH High frequency of horizontal gene transfer in the oceans Science 2010 330 50 10.1126/science.1192243 20929803

[B10] Trobos M Lester CH Olsen JE Frimodt-Moller N Hammerum AM Natural transfer of sulphonamide and ampicillin resistance between Escherichia coli residing in the human intestine J Antimicrob Chemother 2009 63 80 86 18957394 10.1093/jac/dkn437

[B11] Beiko RG Harlow TJ Ragan MA Highways of gene sharing in prokaryotes Proc Natl Acad Sci USA 2005 102 14332 14337 10.1073/pnas.0504068102 16176988 PMC1242295

[B12] Boussau B Gueguen L Gouy M Accounting for horizontal gene transfers explains conflicting hypotheses regarding the position of aquificales in the phylogeny of Bacteria BMC evolutionary biology 2008 8 272 10.1186/1471-2148-8-272 18834516 PMC2584045

[B13] Zhaxybayeva O Swithers KS Lapierre P Fournier GP Bickhart DM DeBoy RT Nelson KE Nesbo CL Doolittle WF Gogarten JP On the chimeric nature, thermophilic origin, and phylogenetic placement of the Thermotogales Proc Natl Acad Sci USA 2009 106 14 5865 5870 10.1073/pnas.0901260106 19307556 PMC2667022

[B14] Roberts AP Mullany P Oral biofilms: a reservoir of transferable, bacterial, antimicrobial resistance Expert Rev Anti Infect Ther 2010 8 1441 1450 10.1586/eri.10.106 21133668

[B15] Gogarten JP Doolittle WF Lawrence JG Prokaryotic evolution in light of gene transfer Mol Biol Evol 2002 19 12 2226 2238 12446813 10.1093/oxfordjournals.molbev.a004046

[B16] Andam CP Williams D Gogarten JP Biased gene transfer mimics patterns created through shared ancestry Proc Natl Acad Sci USA 2010 107 10679 10684 10.1073/pnas.1001418107 20495090 PMC2890805

[B17] Woese CR Olsen GJ Ibba M Soll D Aminoacyl-tRNA synthetases, the genetic code, and the evolutionary process Microbiol Mol Biol Rev 2000 64 202 236 10.1128/MMBR.64.1.202-236.2000 10704480 PMC98992

[B18] Paster BJ Dewhirst FE Phylogenetic foundation of spirochetes J Mol Microbiol Biotechnol 2000 2 341 344 11075904

[B19] Greenberg EP Canale-Parola E Spirochaeta halophila sp. n., a facultative anaerobe from a high-salinity pond Arch Microbiol 1976 110 185 194 10.1007/BF00690227 1015946

[B20] Aksenova H Rainey F Janssen P Zavarzin G Morgan H Spirochaeta thermophila sp. nov., an obligately anaerobic, polysaccharolytic, extremely thermophilic bacterium Int J Syst Bacteriol 1992 42 175 177 10.1099/00207713-42-1-175

[B21] Pohlschroeder M Leschine S Canale-Parola E Spirochaeta caldaria sp. nov., a thermophilic bacterium that enhances cellulose degradation by Clostridium thermocellum Archives Microbiol 1994 161 17 24

[B22] Magot M Fardeau ML Arnauld O Lanau C Ollivier B Thomas P Patel BK Spirochaeta smaragdinae sp. nov., a new mesophilic strictly anaerobic spirochete from an oil field FEMS Microbiol Lett 1997 155 185 191 10.1111/j.1574-6968.1997.tb13876.x 9351200

[B23] Hoover RB Pikuta EV Bej AK Marsic D Whitman WB Tang J Krader P Spirochaeta americana sp. nov., a new haloalkaliphilic, obligately anaerobic spirochaete isolated from soda Mono Lake in California Int J Syst Evol Microbiol 2003 53 815 821 10.1099/ijs.0.02535-0 12807206

[B24] Pikuta EV Hoover RB Bej AK Marsic D Whitman WB Krader P Spirochaeta dissipatitropha sp. nov., an alkaliphilic, obligately anaerobic bacterium, and emended description of the genus Spirochaeta Ehrenberg 1835 Int J Syst Evol Microbiol 2009 59 1798 1804 19578151 10.1099/ijs.0.65862-0

[B25] Droge S Frohlich J Radek R Konig H Spirochaeta coccoides sp. nov., a novel coccoid spirochete from the hindgut of the termite Neotermes castaneus Appl Environ Microbiol 2006 72 392 397 10.1128/AEM.72.1.392-397.2006 16391069 PMC1352290

[B26] Burley SK Petsko GA Aromatic-aromatic interaction: a mechanism of protein structure stabilization Science 1985 229 23 28 10.1126/science.3892686 3892686

[B27] Anderson DE Hurley JH Nicholson H Baase WA Matthews BW Hydrophobic core repacking and aromatic-aromatic interaction in the thermostable mutant of T4 lysozyme Ser 117-- > Phe Protein Sci 1993 2 1285 1290 10.1002/pro.5560020811 8401213 PMC2142442

[B28] Kannan N Vishveshwara S Aromatic clusters: a determinant of thermal stability of thermophilic proteins Protein Eng 2000 13 753 761 10.1093/protein/13.11.753 11161106

[B29] Huang J Gogarten JP Ancient gene transfer as a tool in phylogenetic reconstruction Methods in molecular biology 2009 532 127 139 10.1007/978-1-60327-853-9_7 19271182

[B30] Huang J Xu Y Gogarten JP The presence of a haloarchaeal type tyrosyl-tRNA synthetase marks the opisthokonts as monophyletic Mol Biol Evol 2005 22 2142 2146 10.1093/molbev/msi221 16049196

[B31] Zhaxybayeva O Lapierre P Gogarten JP Genome mosaicism and organismal lineages Trends Genet 2004 20 5 254 260 10.1016/j.tig.2004.03.009 15109780

[B32] Arnold M Evolution through genetic exchange 2006 Oxford, Great Britain: Oxford University Press

[B33] Puigbo P Wolf YI Koonin EV The tree and net components of prokaryote evolution Genome Biol Evol 2010 2 745 756 10.1093/gbe/evq062 20889655 PMC2997564

[B34] Andam CP Gogarten JP Biased gene transfer in microbial evolution Nat Rev Microbiol 2011 9 7 543 555 10.1038/nrmicro2593 21666709

[B35] Wolf YI Aravind L Grishin NV Koonin EV Evolution of aminoacyl-tRNA synthetases--analysis of unique domain architectures and phylogenetic trees reveals a complex history of horizontal gene transfer events Genome Res 1999 9 8 689 710 10447505

[B36] Farahi K Pusch GD Overbeek R Whitman WB Detection of lateral gene transfer events in the prokaryotic tRNA synthetases by the ratios of evolutionary distances method Journal of molecular evolution 2004 58 5 615 631 10.1007/s00239-004-2582-2 15170264

[B37] Novichkov PS Omelchenko MV Gelfand MS Mironov AA Wolf YI Koonin EV Genome-wide molecular clock and horizontal gene transfer in bacterial evolution J Bacteriol 2004 186 19 6575 6585 10.1128/JB.186.19.6575-6585.2004 15375139 PMC516599

[B38] Eriani G Delarue M Poch O Gangloff J Moras D Partition of tRNA synthetases into two classes based on mutually exclusive sets of sequence motifs Nature 1990 347 6289 203 206 10.1038/347203a0 2203971

[B39] Burbaum JJ Schimmel P Structural relationships and the classification of aminoacyl-tRNA synthetases J Biol Chem 1991 266 26 16965 16968 1894595

[B40] Zhaxybayeva O Gogarten JP Cladogenesis, coalescence and the evolution of the three domains of life Trends in Genetics 2004 20 4 182 187 10.1016/j.tig.2004.02.004 15041172

[B41] Fournier GP Huang J Gogarten JP Horizontal gene transfer from extinct and extant lineages: biological innovation and the coral of life Philosophical transactions of the Royal Society of London 2009 364 2229 2239 10.1098/rstb.2009.0033 19571243 PMC2873001

[B42] Frost LS Leplae R Summers AO Toussaint A Mobile genetic elements: the agents of open source evolution Nat Rev Microbiol 2005 3 9 722 732 10.1038/nrmicro1235 16138100

[B43] Olendzenski L Zhaxybayeva O Gogarten JP Seckbach J What's in a Tree?: Does Horizontal Gene Transfer Determine Microbial Taxonomy? Cellular Origin and Life in Extreme Habitats Vol 4: Symbiosis 2001 4 Netherlands: Kluwer Academic Publishers 67 78

[B44] Stern A Mayrose I Penn O Shaul S Gophna U Pupko T An evolutionary analysis of lateral gene transfer in thymidylate synthase enzymes Syst Biol 2010 59 2 212 225 10.1093/sysbio/syp104 20525631 PMC2826268

[B45] Andam CP Gogarten JP Biased gene transfer in microbial evolution Nat Rev Micro 2011 9 7 543 555 10.1038/nrmicro2593 21666709

[B46] Cairns-Smith AG Seven clues to the origin of life 1985 Cambridge, UK: Cambridge University Press

[B47] William J Pragmatism: A New Name for Some Old Ways of Thinking, Lecture IV: The One and the Many 1907 New York: Longmans, Green and Co

[B48] Gribaldo S Brochier C Phylogeny of prokaryotes: does it exist and why should we care? Res Microbiol 2009 160 513 521 10.1016/j.resmic.2009.07.006 19631737

[B49] Valas R Bourne P Save the tree of life or get lost in the woods Biol Direct 2010 5 44 10.1186/1745-6150-5-44 PMC2910001 20594329

[B50] Franklin-Hall L Trashing life's tree Biology and Philosophy 2010 25 4 689 709 10.1007/s10539-010-9219-1

[B51] Bapteste E O'Malley MA Beiko RG Ereshefsky M Gogarten JP Franklin-Hall L Lapointe FJ Dupre J Dagan T Boucher Y Prokaryotic evolution and the tree of life are two different things Biol Direct 2009 4 34 10.1186/1745-6150-4-34 19788731 PMC2761302

[B52] Altschul SF Gish W Miller W Myers EW Lipman DJ Basic local alignment search tool J Mol Biol 1990 215 403 410 2231712 10.1016/S0022-2836(05)80360-2

[B53] Edgar RC MUSCLE: multiple sequence alignment with high accuracy and high throughput Nucl Acids Res 2004 32 5 1792 1797 10.1093/nar/gkh340 15034147 PMC390337

[B54] Guindon S Gascuel O A simple, fast, and accurate algorithm to estimate large phylogenies by maximum likelihood Syst Biol 2003 52 696 704 10.1080/10635150390235520 14530136

[B55] Whelan S Goldman N A general empirical model of protein evolution derived from multiple protein families using a maximum-likelihood approach Mol Biol Evol 2001 18 5 691 699 11319253 10.1093/oxfordjournals.molbev.a003851

[B56] Holder ME Roger A PUZZLEBOOT 1999 Marine Biological Laboratory, Woods Hole, MA

[B57] Schmidt HA Strimmer K Vingron M von Haeseler A TREE-PUZZLE: maximum likelihood phylogenetic analysis using quartets and parallel computing Bioinformatics 2002 18 502 504 10.1093/bioinformatics/18.3.502 11934758

[B58] Felsenstein J PHYLIP Manual v1.03 1993 Berkeley, CA: University Herbarium, University of California

[B59] Ronquist F Huelsenbeck JP MrBayes 3: Bayesian phylogenetic inference under mixed models Bioinformatics 2003 19 1572 1574 10.1093/bioinformatics/btg180 12912839

[B60] Lanave C Preparata G Saccone C Serio G A new method for calculating evolutionary substitution rates Journal of molecular evolution 1984 20 1 86 93 10.1007/BF02101990 6429346

[B61] Abascal F Zardoya R Posada D ProtTest: selection of best-fit models of protein evolution Bioinformatics 2005 21 2104 2105 10.1093/bioinformatics/bti263 15647292

[B62] Posada D jModelTest: phylogenetic model averaging Mol Biol Evol 2008 25 1253 1256 10.1093/molbev/msn083 18397919

[B63] Andam C Fournier G Gogarten J Multilevel populations and the evolution of antibiotic resistance through horizontal gene transfer FEMS Microbiology Reviews 2011 Epub May 2011 10.1111/j.1574-6976.2011.00274.x 21521245

[B64] O'Connor E Shand R Halocins and sulfolobicins: the emerging story of archaeal protein and peptide antibiotics J Ind Microbiol Biotech 2001 28 23 31 10.1038/sj/jim/7000190 11938468

[B65] Fournier GP Gogarten JP Rooting the Ribosomal Tree of Life Mol Biol Evol 2010 27 8 1792 1801 10.1093/molbev/msq057 20194428

[B66] Andam CP Williams D Gogarten JP Natural taxonomy in light of horizontal gene transfer Biol Philos 2010 25 589 602 10.1007/s10539-010-9212-8

[B67] Treangen T Rocha E Horizontal transfer, not duplication, drives the expansion of protein families in prokaryotes PLoS Genetics 2011 7 e1001284 10.1371/journal.pgen.1001284 21298028 PMC3029252

[B68] Huang J Gogarten JP Ancient horizontal gene transfer can benefit phylogenetic reconstruction Trends Genet 2006 22 7 361 366 10.1016/j.tig.2006.05.004 16730850

[B69] Huang J Gogarten P Did an ancient chlamydial endosymbiosis facilitate the establishment of primary plastids? Genome Biol 2007 8 6 R99 10.1186/gb-2007-8-6-r99 17547748 PMC2394758

[B70] Frickey T Lupas AN PhyloGenie: automated phylome generation and analysis Nucleic Acids Res 2004 32 17 5231 5238 10.1093/nar/gkh867 15459293 PMC521674

[B71] Olendzenski L Zhaxybayeva O Gogarten JP Syvanen M, Kado CI Horizontal gene transfer: A new taxonomic principle? Horizontal Gene Transfer 2002 2 New York: Academic Press 427 435

